# Novel, heterozygous, de novo pathogenic variant (c.4963delA: p.Thr1656Glnfs*42) of the *NF1* gene in a Chinese family with neurofibromatosis type 1

**DOI:** 10.1186/s12920-023-01514-x

**Published:** 2023-04-24

**Authors:** Lisha Yang, Jiewen Fu, Jingliang Cheng, Baixu Zhou, Maomei Chen, Songyot Anuchapreeda, Junjiang Fu

**Affiliations:** 1 Department of Medical Technology, Faculty of Associated Medical Sciences, Chiang Mai University, Chiang Mai, 50200 Thailand; 2 Key Laboratory of Epigenetics and Oncology, the Research Center for Preclinical Medicine, Southwest Medical University, 3-319, Zhongshan Rd, Luzhou, Sichuan 646000 China; 3 Department of Obstetrics, the Affiliated Hospital of Southwest Medical University, Luzhou, Sichuan 646000 China; 4 Department of Gynecology and Obstetrics, Guangdong Women and Children Hospital, Guangzhou, Guangdong China; 5 Research Center of Pharmaceutical Nanotechnology, Chiang Mai University, Chiang Mai, 50200 Thailand

**Keywords:** Neurofibromatosis type 1 (NF1), Whole exome-sequencing (WES), Short tandem repeat, De novo pathogenic variant, Frameshift

## Abstract

Neurofibromatosis type 1 (NF1) presents an autosomal dominant, haploinsufficient, and multisystemic disorder with patches of skin café-au-lait spots, lisch nodules in the iris, even tumors in the peripheral nervous system or fibromatous skin. In this study, a Chinese young woman who suffered from NF1 disease with first-trimester spontaneous abortion was recruited. Analysis for whole exome sequencing (WES), Sanger sequencing, short tandem repeat (STR), and co-segregation was carried out. As results, a novel, heterozygous, de novo pathogenic variant (c.4963delA:p.Thr1656Glnfs*42) of the *NF1* gene in the proband was identified. This pathogenic variant of the *NF1* gene produced a truncated protein that lost more than one-third of the NF1 protein at the C-terminus including half of the CRAL-TRIO lipid-binding domain and nuclear localization signal (NLS), thus leading to pathogenicity (ACMG criteria: PVS1 + PM2 + PM2). Analysis for NF1 conservation in species revealed high conservation in different species. Analysis of NF1 mRNA levels in different human tissues showed low tissue specificity, which may affect multiple organs presenting other symptoms or phenotypes. Moreover, prenatal *NF1* gene diagnosis showed both alleles as wild types. Thus, this *NF1* novel variant probably underlays the NF1 pathogenesis in this pedigree, which would help for the diagnosis, genetic counseling, and clinical management of this disorder.

**Supplementary Information:**

The online version contains supplementary material available at 10.1186/s12920-023-01514-x.

## Introduction

Neurofibromatosis type 1 (NF1) (OMIM: 162,200), as an autosomal dominant, haploinsufficient and multisystemic disorder, presented with patches of skin café-au-lait spots, lisch nodules of the iris, tumors of the peripheral nervous system and fibromatous skin [1–4]. At least 60% of NF1 patients develop cancer over their lifetime and almost all have benign cutaneous neurofibromas. The incidence of NF1 disease is 1 in 2,500 to 1 in 3,000 cases worldwide [5]. By linkage analysis, *NF1* gene was mapped to chromosome 17q11.2 [6, 7]. Then, Wallace et al. [8] further characterized a large transcript from the *NF1* gene region of 17q11.2 in three patients carrying NF1 disease. This large transcript was disrupted *NF1* gene expression due to translocations t (17;22) and t (1;17), or a 0.5-kb insertion on NF1 region. This large transcript is the *NF1* gene, called neurofibromin 1 (OMIM: 613,113). Latter more *NF1* gene germline mutations that caused neurofibromatosis type 1 diseases have been identified [9, 10].

The *NF1* gene (NM_001042492.3) is 8,517 bps in coding sequences (CDS) length and encodes 2,839 amino acids in length with predicted molecular mass 319 kDa in weight (NP_001035957.1). This large gene (60 exons and > 300 kilobases (kb) of genomic DNA) has one of the highest rates of spontaneous mutations in the entire human genome. The gene mutations of *NF1* with different types vary from complete gene deletions, insertions, stop, splicing mutations, amino acid substitutions and chromosomal rearrangements, leading the loss of heterozygosity of *NF1* gene function related mainly in neurons, Schwann cells, oligodendrocytes, and leukocytes, thus causing Multisystem abnormities. [11]. Although NF1 is usually fully penetrant by age 5, there is a high degree of variability and unpredictability in disease outcome, even between closely related family members [12]. The “two-hit” hypothesis is one of the most popular etiology, which first proposed by Knudson suppose that some manifestations associated with NF1, such as cognitive problems, resulting from haploinsufficiency of NF1. Others may require a somatic mutation resulting in biallelic NF1 inactivation– as seen in the development of café-au-lait macules (CALMs), neurofibromas, duodenal carcinoid, glomus tumors, bone abnormalities and so on [12]. Spliced transcripts that expressed different NF1 isoforms have been discovered. The NF1 protein functions as a negative mediator of the RAS signal transduction pathway, ERK/MAP pathway, adenylyl cyclase, and cytoskeletal assembly [13, 14]. In the RAS signal transduction pathway, *NF1* gene product directly inhibits RAS activation by converting the active form of GTP-bound RAS to its inactive, GDP-bound state. The end result of RAF/MAPK inactivation is suppression of transcription and cell growth. Thus, neurofibromin deficiency leads to increased RAS signaling which is assumed to be the root cause of NF1 pathology [12]. As a tumor-suppressive gene, *NF1* gene mutations are linked not only to neurofibromatosis type 1 but also to juvenile myelomonocytic leukemia (OMIM: 607,785), familial spinal neurofibromatosis (OMIM:162,210), and Watson syndrome (OMIM: 193,520), etc. [15, 16]. Mutations of the *NF1* gene showed a broad spectrum of clinical characteristics, making it a challenge to detect mutations in NF1, genetics and molecular testing are thus necessary [17–20].

In the current study, we have successfully identified a novel, de novo pathogenic variant (p. Thr1656Glnfs*42) of *NF1* in a Chinese patient with NF1 by whole exome sequencing (WES), Sanger sequencing, and family co-segregation analysis.

## Methods

### NF1 pedigree recruitment, sample collection, and DNA isolation

**Fig. 1:**
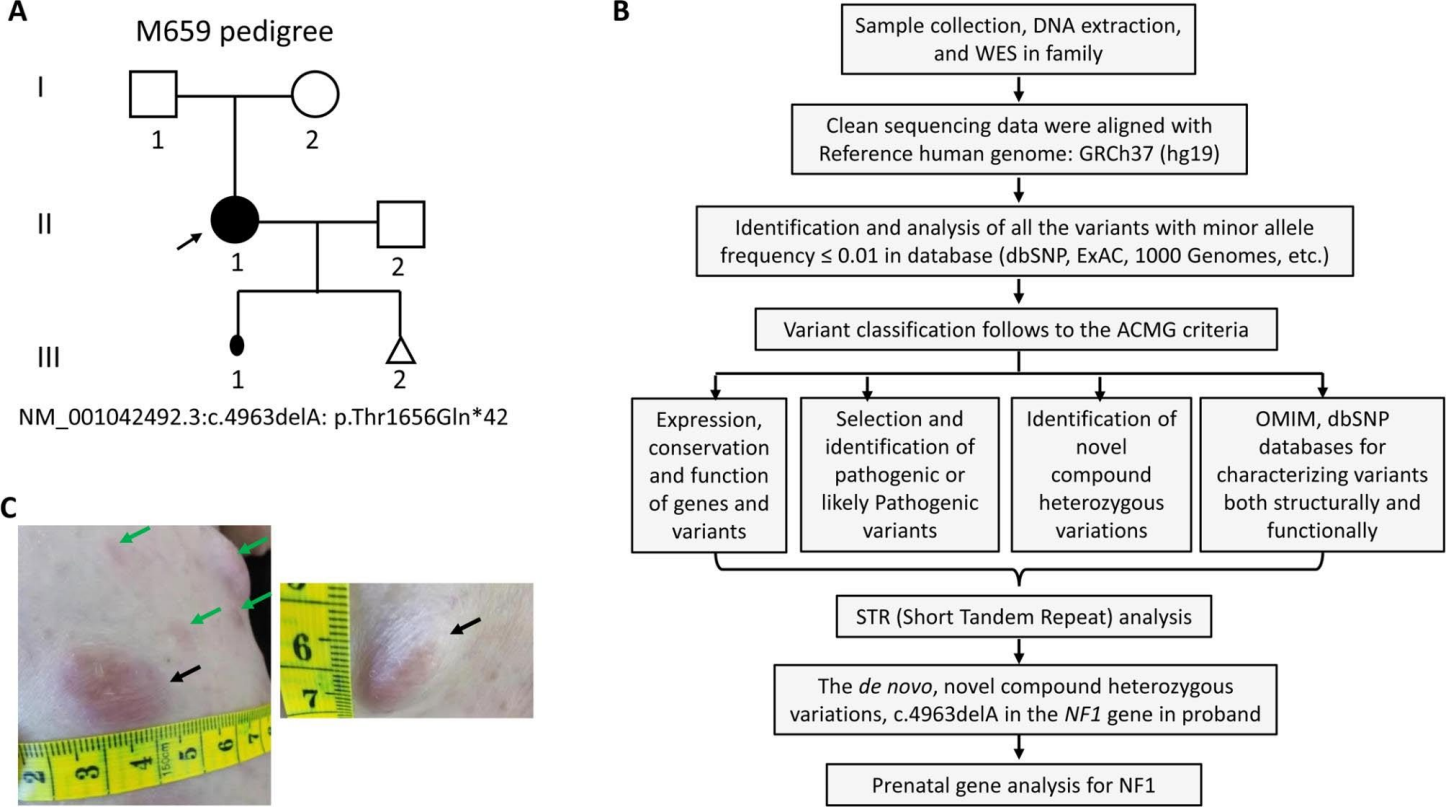
An M659 pedigree with neurofibromatosis type 1. **A**. M659 pedigree with neurofibromatosis type 1. No signs of NF1 individuals are shown as a clear circle (female) or square (male). The filled circle indicates the proband (II: 1, arrow) with the mutation of the *NF1* gene: NM_001042492.3:exon37:c.4963delA:p.Thr1656Glnfs*42. A filled small oval shows an aborted baby. **B**. The detailed variants interpretation pipeline. **C**. The phenotype of neurofibromatosis type 1 in the proband (II:1)

A Chinese female proband with NF1 was recruited and she was first diagnosed at the Dermatology Department in a local county hospital of Gansu Province, in northwest China in the M659 pedigree (Fig. 1A; I:1, M657; I:2, M658; II:1, M659; II:2, M660). The proband’s parents had no clinical manifestation of NF1 while the father was from Anyue of Sichuan Province in southwest China and the mother was from Gansu Province in northwest China. The proband married her husband from Luzhou, a city in Sichuan Province in southwest of China. Peripheral DNA templates from the proband and her family members were isolated with a previous method [21, 22]. DNA from blood samples was taken from healthy individuals (n = 100).

The study was approved by the ethical committee of Southwest Medical University according to the Helsinki Declaration. Written informed consent was obtained from the participants of this family.

### Whole exome sequencing (WES) analysis

WES analysis was conducted on gDNA for the proband M659 (II:1), and for proband parents (M657, I:1; M658, I:2) as described previously [23, 24]. After library preparation, hybridization, PCR amplification, and purification, cluster generation, the Next Generation Sequencing (NGS) was conducted on the Illumina instrument (Illumina, Inc., San Diego, CA). For details, Agilent SureSelect version 6 (Agilent Technologies) was used for capturing sequences. WES reads were aligned to the GRCh37/hg19 through Burrows-Wheeler Aligner software (version 0.59). After that, GATK IndelRealigner was used for local realignment of the Burrows‐Wheeler aligned reads. Then, using the GATK base recalibrator to recalibrate the base quality of the Burrows‐Wheeler aligned reads. The identificatied single‐nucleotide variants (SNV) and insertions or deletions (InDel) have been done by GATK Unified Genotyper. After that, annotation of variants has been done with the Consensus Coding Sequences Database (20,130,630) at the NCBI. Illumina pipeline was used for image analysis and base calling. Indexed primers were used for data fidelity surveillance. SOAP aligner (soap2.21) software was applied to align the clean sequencing reads with human reference genome (hg19). Then, to assemble the consensus sequence and call genotypes in target regions, SOAPsnp (v1.05) software was used. Identified WES variants were selected for data interpretation with minor allele frequency variations and together with their segregation analysis. The function of the variant and their correlation with the disease phenotype were done by OMIM database and previously published literature [25]. Schematic presentation of the detailed and comprehensive data interpretation process is described in Fig. 1B [26–28]. Read coverage per BED region is 99.46% for proband, 99.70% for proband father, and 99.45% for proband mother; read depth per BED region is 144.44 (> 20×, 98.83%) for proband, 134.35 (> 20×, 98.99%) for proband father and 128.85 (> 20×, 98.71%) for proband mother. The pathogenic variant sites were annotated and the annotated file was obtained [29, 30].

### Bioinformatics

Conserved domains from NCBI’s conserved domain database for the NF1 protein (GenBank access no. NP_001035957.1) were searched by the URL (https://www.ncbi.nlm.nih.gov/homologene?Db=homologene&Cmd=Retrieve&list_uids=226) [31–33]. The consensus dataset for RNA-seq data in human tissues was obtained from the Genotype-Tissue Expression (GTEx) project and CAGE data from the FANTOM5 project (https://www.proteinatlas.org/ENSG00000196712-NF1/tissue).

### Sanger verification and segregation analysis

PCR amplification and Sanger sequencing were performed for pathogenic variant verification [34]. Primers were designed by Primer3 program in the *NF1* gene (NF1-4963L: 5’-TCAAAACTGGTCAAATCAATGG-3’; NF1-4963R: 5’-CAAGGTGGCAGCAGGTAGTT-3’). PCR amplification was performed. The PCR conditions: 95 °C for 90 s, 33 cycles of 95 °C for 30 s, 30 s annealing at 60 °C, and 72 °C for 30 s, followed by a final extension at 72 °C for 5 min. The amplified products with 356 bp were then used for Sanger sequencing on an ABI-3500DX sequencer using primer NF1-4963 L.

### Genotyping for STR (short tandem repeat) analysis

Using the AGCU Ex22 kit, the STR genotype was conducted in accordance with the relevant provisions of the Technical Specification for Paternity Appraisal by China (SF/Z JD0105001-2018); STR profiles and the combined paternity index (CPI) were calculated by using the GeneMapper^®^ ID-X 1.5 software [35, 36].

### Prenatal gene analysis for NF1

The amniotic fluid of the fetus was extracted through amniocentesis at 21 weeks of the proband’s pregnancy and then the DNA was extracted as previously reported [37]. Sanger sequencing was performed after PCR amplification using primers NF1-4963 L and NF1-4963R.

## Results

### Clinical diagnosis and characteristics for NF1 proband

The proband was a 34-year-old woman from a non-consanguineous Chinese family (Fig. 1A, II: 1). She was born at full-term delivery with multiple café-au-lait spots scattered on her skin. She claimed a red cutaneous nodule on her back when she was 6 years old and was diagnosed with NF1 after she underwent a nodule biopsy at the age of 10. After puberty, the number of her cutaneous nodules began to increase while café-au-lait spots gradually decreased in number and size. In 2020, due to obvious enlargement of the scalp and facial nodules, several nodules were removed, and a physical examination was carried out. There were many hard cutaneous nodules around her body of different sizes from 0.2−2.0 cm in diameter, with clear boundaries, movably and no tenderness and a small number of subcutaneous nodules. Some light colour café-au-lait spots were on her skin (Fig. 1C; Table 1). In addition to high myopia, the proband did not find abnormalities in bones, blood vessels, central nervous system, hearing and heart. Unfortunately, the proband suffered from first-trimester spontaneous abortion in May, 2021 (Fig. 1A, III: 1), and appeared growth of existing tumors during her pregnancy. However, her parents and husband showed no signs of NF1 (Data not shown).

**Table 1:** The clinical details for M659

Gender	Female
Age (years)	34
Skin	Café-au-lait spots with light colour
Peripheral Nervous System Tumors	Cutaneous Neurofibroma from 0.2-2.0 cm in diameter and a small amount of subcutaneous nodules mainly on her trunk and limbs
Ophthalmic	-
Central Nervous System	-
Musculoskeletal	-
Cardiovascular	-
Central Nervous System Tumors	-
Family History	With no NF1 family history from both of her parents
Pregnancy	Suffered from first-trimester spontaneous abortion with her first pregnancy, and amniocentesis was performed to extract the amniotic fluid of the fetus at 21 gestational weeks of her second pregnancy
Other Tumors	-

### Identifying a de novo heterozygous pathogenic variant (c.4963delA: p.Thr1656Glnfs*42) of the proband with NF1

**Fig. 2:**
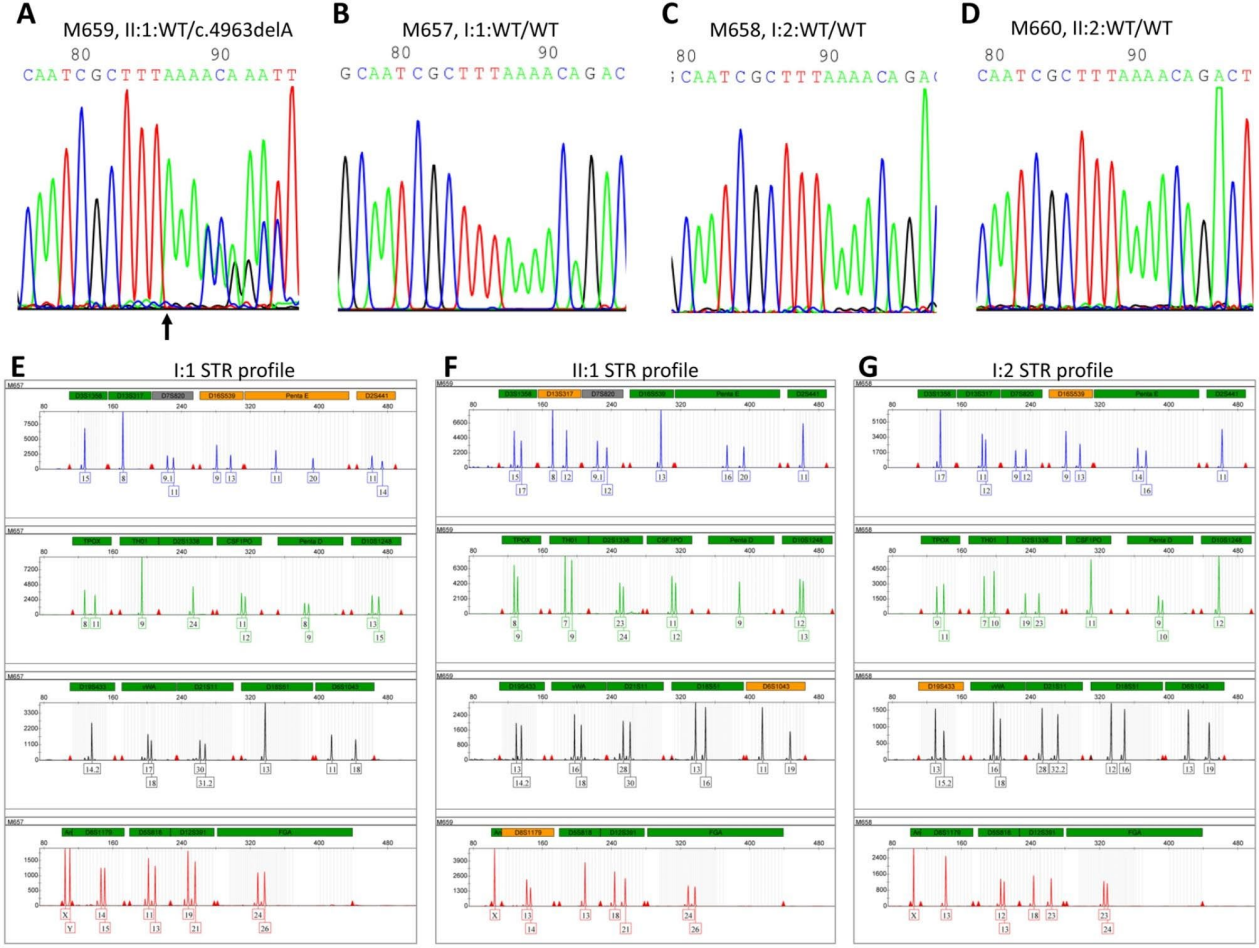
Electropherogram profiles for Sanger sequencing and STR genotypes in the M659 family. **A, B, C**, and **D** indicate the sequenced results in II: 1 (heterozygous mutant type), I: 1 (wild-type), I: 2 (wild-type), II:2 (wild-type) of variant c.4963delA in the *NF1* gene, respectively. The arrows show the mutant position. “WT” indicates wild type. **E ~ G**. Short tandem repeat (STR) genotypes from the family of M659 pedigree. **E**. An electropherogram of STR genotypes from proband’s father I:1. **F**. An electropherogram of STR genotypes from the proband herself II:1. **G**. An electropherogram of STR genotypes from proband’s mother I:1. The “Y” axis indicates the values of RFU (relative fluorescence units), whereas the “X” axis indicates the STR markers for loci

**Fig. 3:**
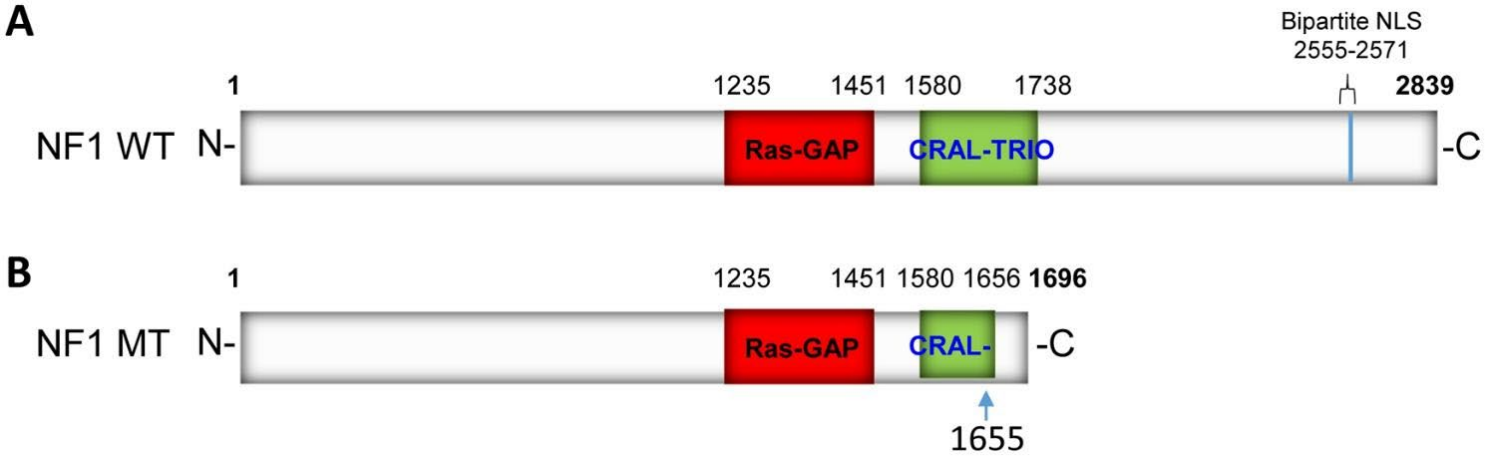
NF1 structure and its mutant form. **A**. The wild-type NF1 domains. **B**. The p.Thr1656Glnfs*42 mutant form. The variant of NF1 is indicated in the CRAL-TRIO domain, where the arrow indicates the mutant position

WES was first performed and then identified a heterozygous pathogenic variant c.4963delA carrying a single nucleotide heterozygous deletion in exon 37 of the *NF1* gene (NM_001042492.3) (chr17: 29652964, the reference human genome version: GRCh37/hg19) for the proband (Fig. 1A, II: 1, Table. 2). This variant leads to amino acid exchanges after a lysine residue at amino acid position 1655 (Lys1655), i.e. p. Thr1656Gln, and a frameshift with another 40 amino acids following a stop codon (p.Thr1656Glnfs*42) in the NF1 protein (NP_001035957.1). Moreover, this mutation was absent in the proband parents of WES data. The c.4963delA mutation was validated by Sanger sequencing (Fig. 2A). This mutation was absent in the 100 ethnic controls. The NF1 protein in humans contains a RAS-GAP domain, a CRAL-TRIO lipid-binding domain at the middle, and nuclear localization signal (NLS) at the NF1 C-terminus (Fig. 3A). The heterozygous pathogenic variant c.4963delA:(p.Thr1656Glnfs*42) losses more than one-third of NF1 of C-terminus including half of CRAL-TRIO lipid-binding domain and NLS (Fig. 3B). Thus, we predicted the protein-level of the pathogenic *NF1* gene was decreased. The loss of function of neurofibromin may therefore remove its regulation in the RAS signal transduction pathway, and lead to uncontrolled cell proliferation. Furthermore, our studies indicated that this *NF1* gene mutation should be pathogenic causing NF1 disease in this proband of the Chinese family. This mutation was excluded in the ExAC, ClinVar, 1000 Human Genome Project, HGMD, and gnomAD databases. Thus, it would be a novel as a pathogenic disease (American College of Medical Genetics and Genomics (ACMG) classification criteria, and it may be PVS1 + PM2 + PM6) [38].

**Table 2:** The results of whole exome sequencing for M659

Gene	Chromosome location	rs No.	Mutant typing	Mutant site	Sequencing depth	Pathogenicity	Diseases
*NF1*	17: 29652964	.	Heterozygous	NM_001042492.3:exon37:c.4963delA:p. Thr1656Glnfs*42	85/89(0.49)	Pathogenic	NF1(AD); Familial spinal neurofibromatosis (AD), etc.

Note: Variant classification follows to the ACMG criteria; "." indicts absence in the databases;" AD "indicates autosomal dominant inheritance; Reference human genome version number: GRCh37 (hg19).

### Segregation of the c.4963delA mutation reveals de novo pathogenic variant of the M659 pedigree

Then Sanger sequencing was conducted for the co-segregation analysis in the members of the M659 pedigree family. This heterozygous pathogenic variant didn’t identify either in the proband’s father (Fig. 2B, M657, I:1) or her mother (Fig. 2C, M658, I:2), i.e., both parents showed *NF1* gene wild type. The proband husband also showed *NF1* gene wild type (Fig. 2D, M660, II:2). Thus, this mutation may not be inherited from the parents but produced a de novo pathogenic variant.

To confirm the paternity, STR analysis was performed using 20 STR markers. The results showed that the STR alleles in this proband (II:1) were inherited from her parents (I:1 and I:2) with a CPI (combined paternity index) of 3.9109 × 10^12^ (> 1 × 10^5^) (Fig. 2E&F&G, Table 3). Thus, segregation and STR analysis reveal a de novo mutation in the M659 pedigree, as a pathogenic disease (American College of Medical Genetics and Genomics (ACMG) classification criteria, PVS1 + PS2 + PM2).

**Table 3:** M659 pedigree STR genotypes

STR marker	I:1 (M657)	II:1(M659)	I:2 (M658)
D3S1358	17	15/17	15
D13S137	11/12	8/12	8
D7S820	9/12	9.1/12	9.1/11
D16S539	9/13	13	9/13
Penta E	14/16	16/20	11/20
D2S441	11	11	11/14
TPOX	9/11	8/9	8/11
TH01	7/10	7/9	9
D2S1338	19/23	23/24	24
CSF1PO	11	11/12	11/12
Penta D	9/10	9	8/9
D10S1248	12	12/13	13/15
D19S433	13/15.2	13/14.2	14.2
vWA	16/18	16/18	17/18
D21S11	28/32.2	28/30	30/31.2
D18S51	12/16	13/16	13
D6S1043	13/19	11/19	11/18
D8S1179	13	13/14	14/15
D5S818	12/13	13	11/13
D12S391	18/23	18/21	19/21
FGA	23/24	24/26	24/26
AMEL	X	X	X/Y

### Analysis for NF1 conservation in species and its expressions in tissues

Analysis for NF1 conservation in species revealed that it is highly conserved in chimpanzees, Rhesus monkeys, dogs, rats, mice, chickens, frogs, zebrafish, fruit flies, and mosquitoes, of different species (Supplementary Fig. 1A). All of them have RAS-GAP domain and CRAL-TRIO lipid-binding domain, demonstrating partially deletion of CRAL-TRIO domain in the proband should cause NF1 disease in this family. Analysis for NF1 mRNA expressions from RNA data in different human tissues found that NF1 mRNA showed low tissue specificity, detected mainly in the brain, colon, peripheral nerve, lung, muscle, etc. (Supplementary Fig. 1B).

### The results for prenatal gene analysis for NF1

**Fig. 4:**
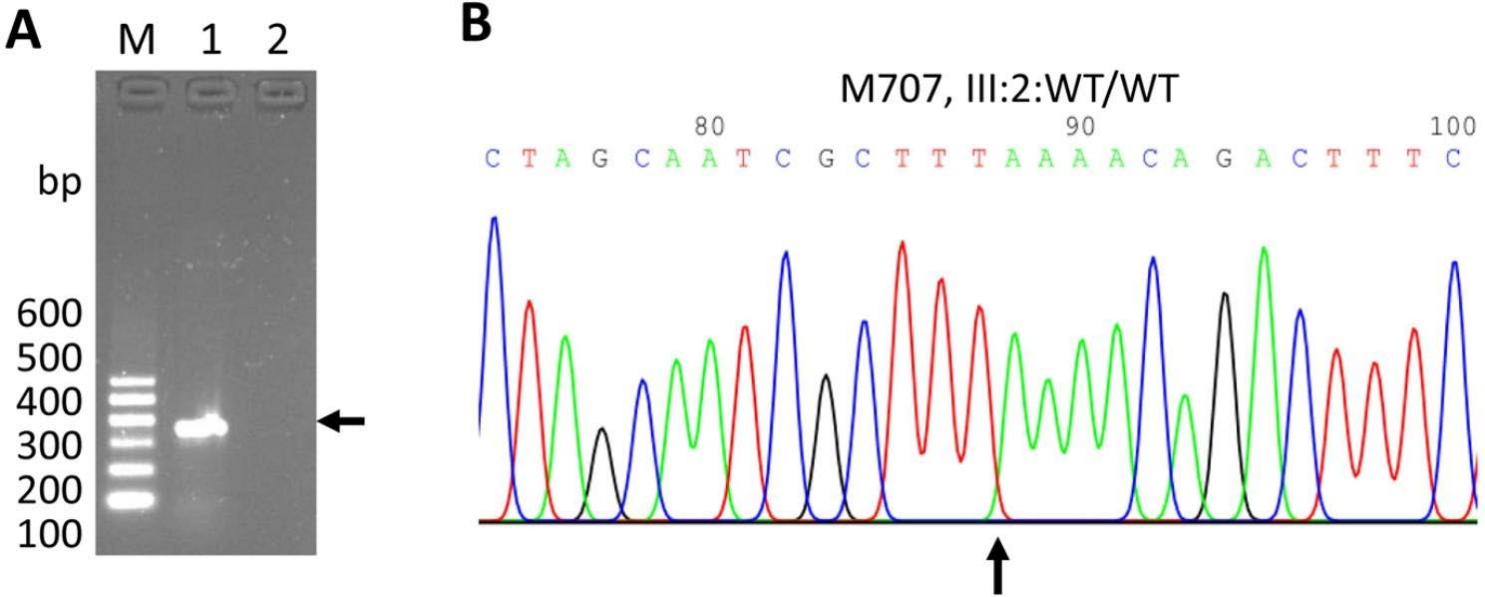
Electropherogram profile for Sanger sequencing in the fetus. **A**. PCR products for the M707 fetus. “M” indicates the DNA ladder with size; “1” indicates the PCR products from M707 fetus (arrow); “2” indicates no PCR products. **B**. Electropherogram profile for Sanger sequencing in M707 fetus

We took Type B ultrasound examination before the amniocentesis showing a singleton, live fetus (data not shown). The Sanger sequencing of the amplified PCR products of amniotic fluid DNA of the fetus revealed the wild type alleles of the *NF1* gene (Fig. 4, M707, III:2), demonstrating that this baby inherited her mother wild type allele, instead of mutant *NF1*. Additionally, the baby showed no NF1 symptoms at five months old.

## Discussion

We recruited a pregnant young woman with NF1 in this study, and identified a novel, de novo, heterozygous pathogenic variant c.4963delA: (p.Thr1656Glnfs*42), which produced a truncated protein causing NF1 pathogenic in this proband of the Chinese family. The heterozygous pathogenic variant c.4963delA:(p.Thr1656Glnfs*42) causes a loss of half of the CRAL-TRIO lipid-binding domain and NLS, thus should cause NF1 disease in this proband. *NF1* gene was reported highly conserved in chimpanzees, Rhesus monkeys, dogs, rats, mice, chickens, frogs, zebrafish, fruit flies, and mosquitoes. Moreover, all of them exhibited RAS-GAP domain and CRAL-TRIO lipid binding domain that demonstrates partial deletion of CRAL-TRIO domain in the proband leading to NF1 disease in this family. Analysis of NF1 mRNA expressions in different human tissues found that NF1 mRNA showed low tissue specificity, mainly detected in the brain, colon, peripheral nerve, lung, muscle, etc., which is not fully consistent with the previous reports [11]. Thus, this mutation may affect multiple organs, leading to the further developing other diseases, symptoms, or phenotypes and guiding our clinical management and genetic counseling.

Dugoff and Sujansky (1996) have reported the outcomes for 247 pregnancies in 105 NF1 women [39]. Of these 247 pregnancies, 44 cases resulted in first-trimester spontaneous abortions. 80% of the women experienced either the appearance of new neurofibromas, the growth of existing tumors, or both. 33% of these women exhibited a decrease in tumor size in the postpartum period. Only 18% of the women presented no tumor size changes and no tumorigenesis during pregnancy. Plexiform neurofibromas are thought to be congenital in origin and occur in about 50% of patients. Plexiform neurofibromas can grow quite large and are estimated to have a 10–30% risk of malignant transformation into a malignant peripheral nerve sheath tumor [40]. For this M659 proband, who also experienced the first-trimester spontaneous abortion, appeared growth of existing tumors during her pregnancy in our current study. In a Danish population-based cohort study, Kenborg (2022) recently reported women with NF1 experienced more spontaneous abortions and stillbirths [25]. The proportion of pregnancies that resulted in a live birth was 63% (783/1252) among NF1 women and 68% (8432/12 465) among the comparisons. The proportions of stillbirths (PR 2.83; 95% CI 1.63 to 4.93) and spontaneous abortions (PR 1.40; 95% CI 1.09 to 1.79) were increased in women with NF1 [25]. However, the reason for the abortion situation is unknown. Pregnant women with NF1 showed an increasing situation of complications including hypertensive disorders that might cause by pheochromocytoma [41].

In previous study, Santasree Banerjee and his co-workers (2017) presented a clinical molecular study of four Chinese probands with NF1 from four unrelated families, showing extreme phenotypic variation with rare phenotype, tibial pseudarthrosis, and anemia [42]. Yao and his colleagues (2019) reported 68 Chinese Neurofibromatosis 1 Children. Pathogenic or likely pathogenic NF1 variants were detected in 71.6% (68/95) of patients; 20 pathogenic variants were not previously reported, indicating that Chinese NF1 patients are still understudied. Parental Sanger sequencing confirmation revealed 77.9% of de novo variants, a percentage that was much higher than expected. The presence of a higher number of NF1-related features at young ages was correlated with positive diagnostic findings [43].

Prenatal diagnosis acted as an effective means of examination in NF1 families. A young girl with NF1 was recruited by Bei Liu et al. [44], and was diagnosed to carry de novo, heterozygous pathogenic variant c.1260 + 4 A > T in *NF1* gene, while her patents contained wild type genotype. But the paternity of this pedigree didn’t confirm. Prenatal diagnosis was carried out at the 20 weeks of gestation on her mother’s second pregnancy, and the baby also showed wild type genotype. Aurélia Gruber et al. [45].performed non-invasive prenatal diagnosis (NIPD) to check four couples at risk of transmitting paternal *NF1* gene mutations between 8 and 15 weeks of gestation, which were in parallel to conventional invasive diagnosis. They designed specific hydrolysis probes to detect the paternal mutation and to assess the presence of cell-free fetal DNA by ddPCR. Despite NIPT possesses high accuracy, invasive prenatal diagnosis remains the gold standard.

As we discussed previously, five possibilities with discordant segregation are existed, including no paternity or errors in sampling, de novo mutation, heterozygous micro-deletion, and uniparental disomy (UPD) [46]. Through homozygosity mapping and STR analysis, a previous study had identified an unusual homozygous mutation of Usher syndrome type IIA pedigree that originated from maternal UPD [46]. In the current study, segregation and STR analysis revealed a de novo pathogenic variant in this M659 pedigree. Genetic counseling and clinical management for these families’ NF1 symptoms should conduct in the possible event that an unaffected individual can affect pathogenic offspring.

Prenatal gene diagnosis was conducted by Sanger sequencing of the DNA of amniotic fluid of the fetus that showed the wild type of the *NF1* gene. The results demonstrate that this baby inherits her mother wild type allele, not mutant *NF1*. The baby showed no NF1 symptoms at five months old. However, further following up should be conducted to monitor’s the baby development.

## Conclusions

We successfully identified a novel, de novo, heterozygous frameshift pathogenic variant in the *NF1* gene, which would cause NF1 disorder in the Chinese family. Next-generation sequencing (NGS) including WES [47] and STR analysis [48] are useful for genetic diagnosis, which help to elucidate the molecular pathogenesis of NF1 disease and to contribute the diagnosis, genetic counseling, and clinical management of this disorder.

## Electronic supplementary material

Below is the link to the electronic supplementary material.

Supplementary Material 1

Supplementary Material 2

## Data Availability

The variant has been submitted to Clinvar under accession number: VCV000566482.10. Other data used for the analyses of this study are available from the corresponding authors upon reasonable request.
