# Supplementary material for: Novel, heterozygous, de novo pathogenic variant (c.4963delA: p.Thr1656Glnfs*42) of the NF1 gene in a Chinese family with neurofibromatosis type 1
Source: BMC Med Genomics. 2023 Apr 24;16:85. doi: 10.1186/s12920-023-01514-x (PMC10123994; doi:10.1186/s12920-023-01514-x)
Supplement: Supplementary file 1 — Supplementary Material 1 [file 12920_2023_1514_MOESM1_ESM.pptx]

## Slide 1
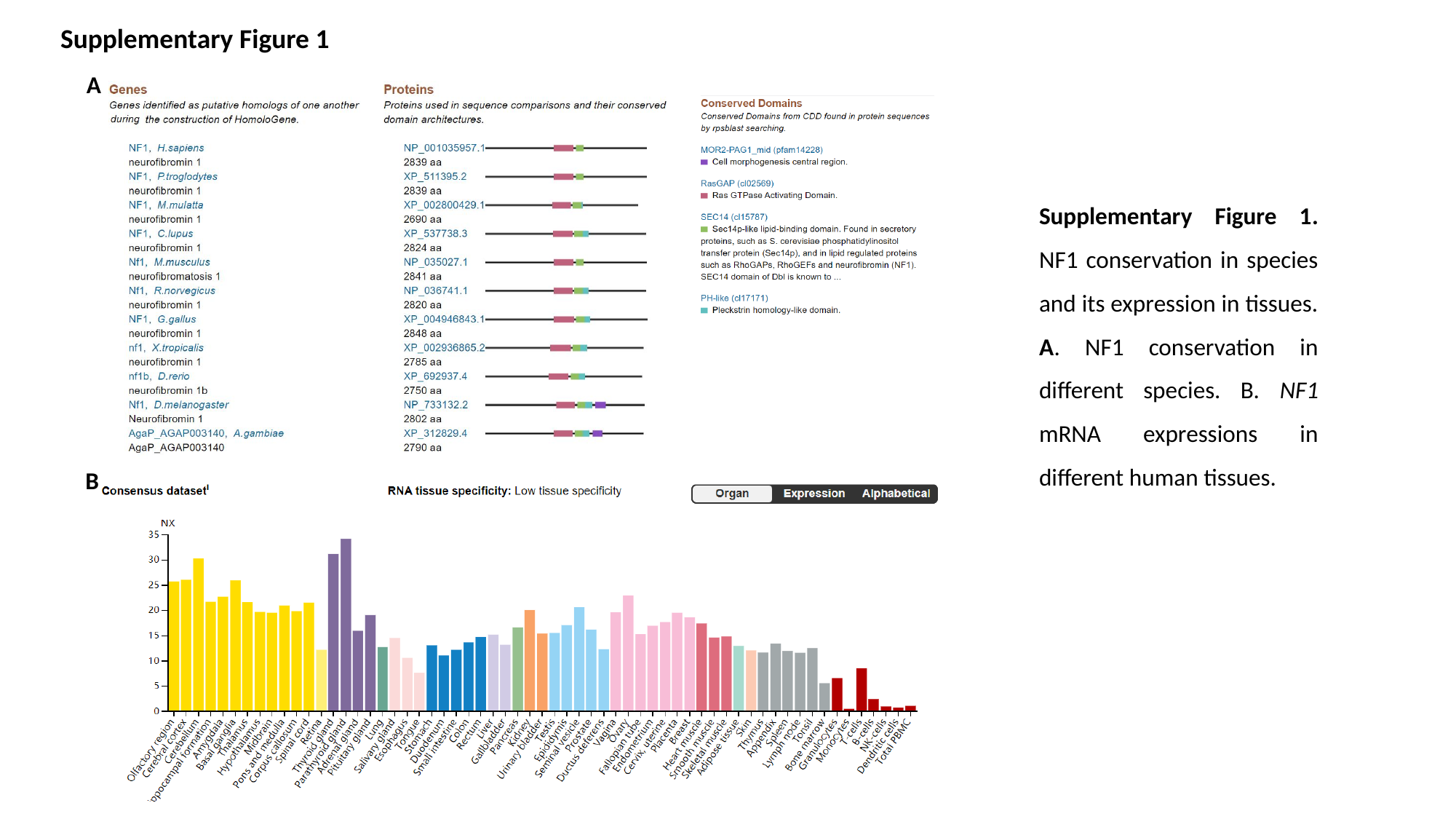

Supplementary Figure 1
A
B
Supplementary Figure 1. NF1 conservation in species and its expression in tissues. A. NF1 conservation in different species. B. NF1 mRNA expressions in different human tissues.
