# Supplementary figures and images for: Novel, heterozygous, de novo pathogenic variant (c.4963delA: p.Thr1656Glnfs*42) of the NF1 gene in a Chinese family with neurofibromatosis type 1
Source: BMC Med Genomics. 2023 Apr 24;16:85. doi: 10.1186/s12920-023-01514-x (PMC10123994; doi:10.1186/s12920-023-01514-x)

**Figure 4**

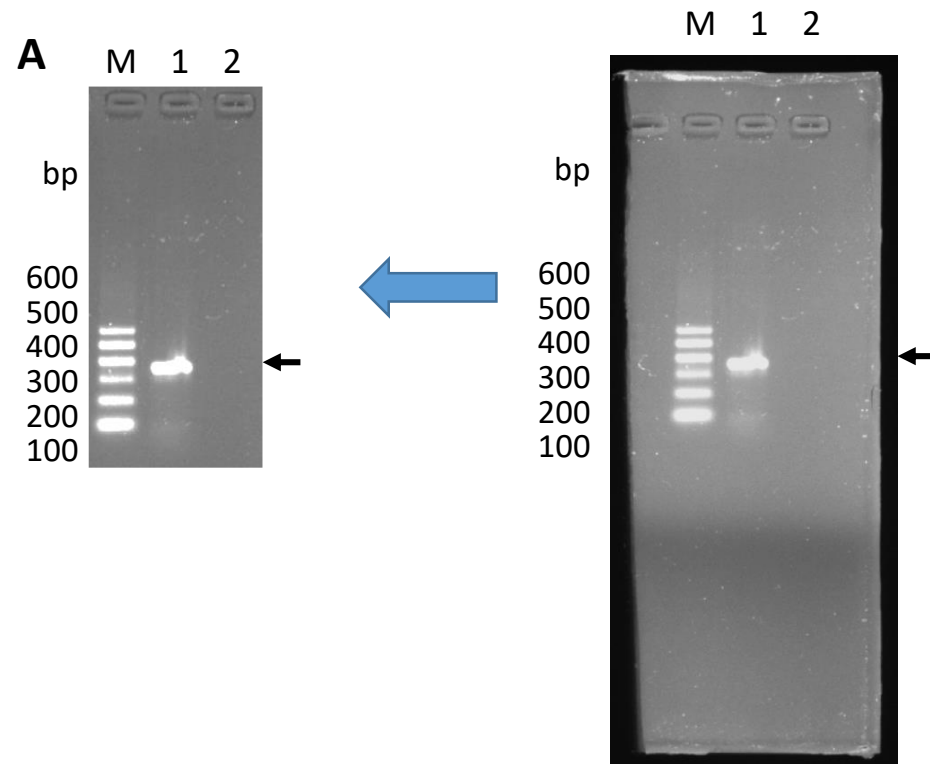

Supplement: Supplementary file 2 — Supplementary Material 2 [file 12920_2023_1514_MOESM2_ESM.pdf]
